# Survival and Treatment Patterns in Stage II to III Esophageal Cancer

**DOI:** 10.1001/jamanetworkopen.2024.40568

**Published:** 2024-10-21

**Authors:** Won Jin Jeon, Daniel Park, Farris Al-Manaseer, Yi-Jen Chen, Jae Y. Kim, Bo Liu, Shengyang Wu, Dani Castillo

**Affiliations:** 1 Division of Medical Oncology and Hematology, Department of Internal Medicine, Loma Linda University, Loma Linda, California; 2 Department of Internal Medicine, University of California, San Francisco-Fresno, Fresno; 3 Department of Internal Medicine, Loma Linda University, Loma Linda, California; 4 Department of Radiation Oncology, City of Hope Medical Center, Duarte, California; 5 Division of Thoracic Surgery, Department of Surgery, City of Hope Medical Center, Duarte, California; 6 Department of Diagnostic Radiation, City of Hope Medical Center, Duarte, California; 7 Department of Medical Oncology and Therapeutics Research, City of Hope Medical Center, Duarte, California

## Abstract

**Importance:**

Existing clinical trials favor neoadjuvant chemoradiation therapy (NCRT) followed by surgery alone for locally advanced esophageal cancer (EC) and perioperative chemotherapy as the preferred modality for esophageal adenocarcinoma (EAC). However, it is unclear whether these trial findings are reflected in the patterns of care and survival outcomes among patients in the clinical setting.

**Objective:**

To investigate survival outcomes in the clinical setting among patients with EC after various treatment modalities.

**Design, Setting, and Participants:**

This retrospective cohort study examined data from the National Cancer Database maintained by the American College of Surgeons and focused on patients with clinical stage II or III EC, excluding those with gastroesophageal junction cancer, who underwent trimodality therapy (NCRT followed by esophagectomy), definitive chemoradiation therapy (DCRT), radiotherapy (RT) alone, or perioperative chemotherapy from January 2006 to December 2020. Analyses were conducted from December 2023 to August 2024.

**Exposures:**

Perioperative chemotherapy, trimodality therapy, DCRT, and single-modality RT.

**Main Outcomes and Measures:**

A Cox proportional hazards regression model was used to compare overall survival (OS) between treatment groups in the entire cohort, among patients with squamous cell carcinoma or adenocarcinoma, and among those older than 65 years. Landmark survival analysis at 6 months was performed to reduce survivorship bias.

**Results:**

The study included 57 116 patients (median age, 64 [IQR, 57-72] years; 45 410 [79.5%] male); 21 619 patients (37.9%) received trimodality therapy, 32 493 (57.1%) received DCRT, 2692 (4.7%) received single-modality RT, and 312 (0.5%) received perioperative chemotherapy. In the overall study population, 37 698 patients (66.0%) had EAC, and of the 312 patients that received perioperative chemotherapy, 283 (90.7%) had EAC. In adjusted survival analysis, perioperative chemotherapy (adjusted hazard ratio [AHR], 0.33; 95% CI, 0.28-0.39; *P* <.001) and trimodality therapy (AHR, 0.45; 95% CI, 0.44-0.46; *P* < .001) were associated with improved OS compared with DCRT. In contrast, RT alone was associated with worse outcomes compared with DCRT (AHR, 1.37; 95% CI, 1.30-1.45; *P* < .001). The median OS for perioperative chemotherapy of 66.2 months (95% CI, 43.1-111.9 months; *P* < .001) was longer compared with that for DCRT alone (18.1 months; 95% CI, 17.8-18.4 months; *P* < .001). Trimodality therapy was associated with a median OS of 43.9 months (95% CI, 42.8-45.5 months; *P* < .001), which was shorter than that for perioperative chemotherapy but improved compared with DCRT and RT alone, which was associated with a median OS of 13.5 months (95% CI, 12.8-14.0 months; *P* < .001). In the subgroup of patients older than 65 years, those who received perioperative chemotherapy had longer median OS (56.7 months; 95% CI, 36.4-115.2 months; *P* < .001) compared with those receiving other treatment modalities (eg, trimodality therapy: 40.1 months; 95% CI, 38.1-42.0 months; *P* < .001). Patients who received RT alone had the worst median OS (13.6 months; 95% CI, 12.8-14.4 months; *P* < .001).

**Conclusions and Relevance:**

In this cohort study of patients with stage II to III EC, trimodality therapy was associated with improved OS compared with DCRT or RT alone for locally advanced EC and perioperative chemotherapy was associated with improved OS for adenocarcinoma.

## Key Points

**Question:**

In clinical settings, are varying treatment modalities associated with different survival outcomes among patients with locally advanced esophageal cancer (EC)?

**Findings:**

In this cohort study of 57 116 patients with locally advanced EC, use of neoadjuvant chemoradiation with surgical resection was associated with improved overall survival compared with definitive chemoradiation (DCRT) or radiotherapy alone.

**Meaning:**

The findings suggest that neoadjuvant chemoradiation with surgical resection is associated with improved survival compared with DCRT or radiotherapy alone for locally advanced EC.

## Teaser

This cohort study examines the association of neoadjuvant chemoradiation with surgical resection, definitive chemoradiation, radiotherapy alone, and perioperative chemotherapy with overall survival among patients with stage II to III esophageal cancer.

## Introduction

Esophageal cancer (EC) remains a significant clinical challenge, with mortality rates ranking ninth among all cancers globally.^1^ Based on histologic features, EC is predominantly classified as esophageal squamous cell carcinoma (ESCC) and esophageal adenocarcinoma (EAC), with ESCC accounting for the majority of cases worldwide and approximately 40% in the US. Despite advances in treatment modalities and literature^2^ demonstrating the efficacy of perioperative chemotherapy in the management of EAC as well as trimodality therapy in managing EC, use of EC treatment modalities and outcomes in the clinical setting are not well defined.

Key studies, such as those by Stahl et al^3^ and Bedenne et al,^4^ demonstrated that incorporating esophagectomy into chemoradiation therapy (CRT) enhanced local control in ESCC. The landmark CROSS trial^5^ and its subsequent 10-year follow-up^6^ found that trimodality therapy—comprising concurrent radiation, carboplatin, and paclitaxel—significantly improved overall survival (OS) compared with surgery alone for EC. The Neo-AEGIS trial,^7^ which compared perioperative chemotherapy with the CROSS trimodality therapy for EAC,^5^ found no significant differences in 3-year survival or quality of life; however, the Neo-AEGIS trial was underpowered and prematurely terminated due to slow accrual and early futility with statistics modified to a smaller sample size. Finally, the ESOPEC trial^8^ concluded that perioperative fluorouracil, leucovorin, oxaliplatin, and docetaxel (FLOT) improved survival among patients with resectable EAC compared with the CROSS trimodality therapy.^5^

Perioperative chemotherapy followed by surgery has emerged as the superior treatment modality for EAC. Although, the Neo-AEGIS trial^7^ demonstrated a clinical equipoise between perioperative chemotherapy and the CROSS trimodality therapy. Nonoperative therapy, particularly for ESCC, involves CRT due to its higher rate of complete response.^5,9^ Radiotherapy (RT) alone is generally considered a palliative treatment.

Our study aimed to evaluate the clinical use of and outcomes associated with different treatment modalities for EC in the clinical setting. We assessed survival outcomes associated with perioperative chemotherapy, trimodality therapy, and RT alone compared with definitive CRT (DCRT).

## Methods

### Data

This retrospective cohort study used data from the National Cancer Database (NCDB), a hospital-based registry of cancer outcomes established collaboratively by the American Cancer Society and the American College of Surgeons Commission on Cancer (COC). The NCDB aggregates data from a wide array of sources, including teaching hospitals, community cancer centers, and Veterans Affairs hospitals across 49 US states and Puerto Rico. Participation in reporting to the NCDB is restricted to COC-approved hospitals, which capture an estimated 70% of all new cancer diagnoses in the US. The NCDB Participant User Data File, a Health Insurance Portability and Accountability Act–compliant repository of deidentified patient-level data submitted to the COC NCDB, served as the primary dataset for this analysis. Given the deidentification of patient information in the Participant User Data File, this analysis was exempt from institutional review board approval. Our study protocol adhered to 45 CFR §46 guidelines, using data that lacked personal identifiers from COC-accredited facilities. As part of our reporting standards, we followed the Strengthening the Reporting of Observational Studies in Epidemiology (STROBE) guideline.

### Cohort

This retrospective cohort study included patients with T2 to T4 or node-positive esophageal carcinoma treated from January 2006 to December 2020. The exclusion criteria were gastroesophageal junction tumors, prior cancer, carcinoma in situ, T1/M1 disease, and receipt of an unknown type of therapy.

### Variables

Demographic variables consisted of age, sex, race, and ethnicity. Race, ethnicity, and all other variables were classified based on the NCDB. Race and ethnicity were included in this analysis because previous studies^10^ have highlighted significant differences in survival outcomes based on race and ethnicity. These data were reported as listed in the NCDB as Black; East Asian, South Asian, or Pacific Islander; Hispanic; White; and other (not further specified in the NCDB). Clinical parameters included Charlson-Deyo Comorbidity Index (CDCI) score (to assess performance status),^11^ histologic subtype, grade, and stage. Treatment-related variables comprised number of positive lymph nodes, treatment facility type, distance from treatment facility to zip code of residence, insurance status, and treatment modality. The treatment modality variable was categorized as perioperative chemotherapy, DCRT, neoadjuvant CRT (NCRT) followed by esophagectomy (hereafter, trimodality therapy), and single-modality RT.

### Outcomes

We compared OS between treatment groups (perioperative chemotherapy, trimodality therapy, DCRT, and RT alone) in the entire cohort. Analyses also compared OS in subgroups of patients with ESCC, those with EAC, and those older than 65 years, who are not well represented in clinical trials.

### Statistical Analysis

Clinicodemographic and pathologic variables were extracted from the database and compared using the χ^2^ test. Univariate Cox proportional hazards regression analyses were performed to assess variables associated with OS. Variables with *P* < .05 on the univariate model that had more than 20 events per variable were included in the multivariate model. In this analysis, the starting point (time 0) was defined as the date of diagnosis as recorded in the database. A landmark survival analysis was performed by excluding patients with less than 6 months of follow-up to address survivorship and immortal time bias, ensuring that only those who survived long enough to potentially complete trimodality therapy or perioperative chemotherapy were included. Median follow-up was estimated by the method of Schemper and Smith.^12^ All analyses were conducted from December 2023 to August 2024 using R, version 4.1.2 (R Project for Statistical Computing). All tests were 2-sided with *P* < .05 as the threshold for significance.

## Results

### Patient Characteristics

This study identified 57 116 patients (median age, 64 [IQR, 57-72] years) diagnosed with locally advanced EC. Patient characteristics are detailed in Table 1. Overall, 30 389 patients (53.2%) in the overall cohort were older than 65 years, and only 3814 (6.7%) were younger than 50 years. The cohort consisted of 11 706 female patients (20.5%) and 45 410 male patients (79.5%). There were 4954 Black patients (8.7%); 1366 East Asian, South Asian, or Pacific Islander patients (2.4%); 1938 Hispanic patients (3.4%); 48 071 White patients (84.2%); and 787 patients (1.4%) classified as other race and ethnicity.

**Table 1.** Baseline Characteristics of Patients Receiving RT Alone, DCRT Alone, NCRT and Surgery, or Perioperative Chemotherapy

Characteristic	Patients, No. (%)^a^				
	Overall (N = 57 116)	RT alone (n = 2692)	DCRT alone (n = 32 493)	NCRT and surgery (n = 21 619)	Perioperative chemotherapy (n = 312)
Age, y					
<50	3814 (6.7)	92 (3.4)	1708 (5.3)	1978 (9.1)	36 (11.5)
50-65	22 913 (40.1)	685 (25.4)	11 538 (35.5)	10 536 (48.7)	154 (49.4)
≥65	30 389 (53.2)	1915 (71.1)	19 247 (59.2)	9105 (42.1)	122 (39.1)
Sex					
Female	11 706 (20.5)	739 (27.4)	7605 (23.4)	3316 (15.3)	46 (14.7)
Male	45 410 (79.5)	1953 (72.5)	24 888 (76.6)	18 303 (84.7)	266 (85.3)
Race and ethnicity					
Black	4954 (8.7)	375 (13.9)	3751 (11.5)	816 (3.8)	12 (3.8)
East Asian, South Asian, or Pacific Islander	1366 (2.4)	77 (2.9)	912 (2.8)	367 (1.7)	10 (3.2)
Hispanic	1938 (3.4)	103 (3.8)	1190 (3.7)	625 (2.9)	20 (6.4)
White	48 071 (84.2)	2089 (77.6)	26 165 (80.5)	19 553 (90.4)	264 (84.6)
Other^b^	787 (1.4)	48 (1.8)	475 (1.5)	258 (1.2)	6 (1.9)
CDCI score					
0	40 788 (71.4)	1821 (67.6)	23 104 (71.1)	15 637 (72.3)	226 (72.4)
1	11 059 (19.4)	519 (19.3)	6085 (18.7)	4387 (20.3)	68 (21.8)
2	3264 (5.7)	192 (7.1)	1975 (6.1)	1083 (5.0)	14 (4.5)
3	2005 (3.5)	160 (5.9)	1329 (4.1)	512 (2.4)	4 (1.3)
Histologic subtype					
Adenocarcinoma	37 698 (66.0)	1427 (53.0)	18 391 (56.6)	17 597 (81.4)	283 (90.7)
ESCC	16 661 (29.2)	1079 (40.1)	12 355 (38.0)	3215 (14.9)	12 (3.8)
Not adenocarcinoma or ESCC	2757 (4.8)	186 (6.9)	1747 (5.4)	807 (3.7)	17 (5.4)
Grade					
Well differentiated	2304 (4.0)	99 (3.7)	1284 (4.0)	916 (4.2)	5 (1.6)
Moderately differentiated	19 757 (34.6)	848 (31.5)	11 000 (33.9)	7780 (36.0)	129 (41.3)
Poorly differentiated	23 138 (40.5)	1022 (38.0)	12 818 (39.4)	9155 (42.3)	143 (45.8)
Unknown	11 914 (20.9)	723 (26.9)	7389 (22.7)	3767 (17.4)	35 (11.2)
Facility type					
Academic or research	22 351 (39.1)	899 (33.4)	10 950 (33.7)	10 329 (47.8)	173 (55.4)
Community	3851 (6.7)	227 (8.4)	2700 (8.3)	914 (4.2)	10 (3.2)
Comprehensive community cancer program	19 762 (34.6)	1030 (38.3)	12 511 (38.5)	6135 (28.4)	86 (27.6)
Integrated network cancer program	10 577 (18.5)	524 (19.5)	6091 (18.7)	3929 (18.2)	33 (10.6)
Unknown	575 (1.0)	12 (0.4)	241 (0.7)	312 (1.4)	10 (3.2)
Distance to facility, km					
<16	29 533 (51.7)	1617 (60.1)	18 406 (56.6)	9374 (43.4)	136 (43.6)
16-31	10 035 (17.6)	462 (17.2)	5734 (17.6)	3779 (17.5)	60 (19.2)
32-79	10 248 (17.9)	420 (15.6)	5385 (16.6)	4377 (20.2)	66 (21.2)
80-159	3965 (6.9)	128 (4.8)	1662 (5.1)	2149 (9.9)	26 (8.3)
≥160	3335 (5.8)	65 (2.4)	1306 (4.0)	1940 (9.0)	24 (7.7)
Insurance status					
Private	19 726 (34.5)	465 (17.3)	8695 (26.8)	10 401 (48.1)	165 (52.9)
Medicare	28 224 (49.4)	1721 (63.9)	17 694 (54.5)	8696 (40.2)	113 (36.2)
Medicaid	4775 (8.4)	249 (9.2)	3153 (9.7)	1358 (6.3)	15 (4.8)
Uninsured	1558 (2.7)	95 (3.5)	1038 (3.2)	417 (1.9)	8 (2.6)
Other government	1604 (2.8)	109 (4.0)	994 (3.1)	496 (2.3)	5 (1.6)
Unknown	1229 (2.2)	53 (2.0)	919 (2.8)	251 (1.2)	6 (1.9)

Abbreviations: CDCI, Charlson-Deyo Comorbidity Index; DCRT, definitive chemoradiation therapy; ESCC, esophageal squamous cell carcinoma; NCRT, neoadjuvant CRT; RT, radiotherapy.

^a^ *P* < .001 for all comparisons.

^b^ Not further specified in the National Cancer Database.

In the entire cohort, 22 351 patients (39.5%) were seen in an academic or research institute, 19 762 (34.6%) in a comprehensive community cancer program, 10 577 (18.5%) in an integrated network cancer program, and 3851 (6.7%) in community center programs. Overall, 39 568 patients (69.3%) traveled within 31 km to their treatment center. A total of 19 726 patients (34.5%) had private insurance, 28 224 (49.4%) had Medicare, and the remaining patients had a combination of Medicaid or other government insurance or were uninsured.

### Tumor Characteristics

Most patients had EAC (37 698 [66.0%]), 16 661 (29.2%) had ESCC, and 2757 (4.8%) did not have ESCC or EAC. Tumors were classified as well differentiated in 2304 patients (4.0%), moderately differentiated in 19 757 (34.6%), poorly differentiated in 23 138 (40.5%), and unknown in 11 914 (20.9%).

### Treatment Modalities

**Figure 1.**
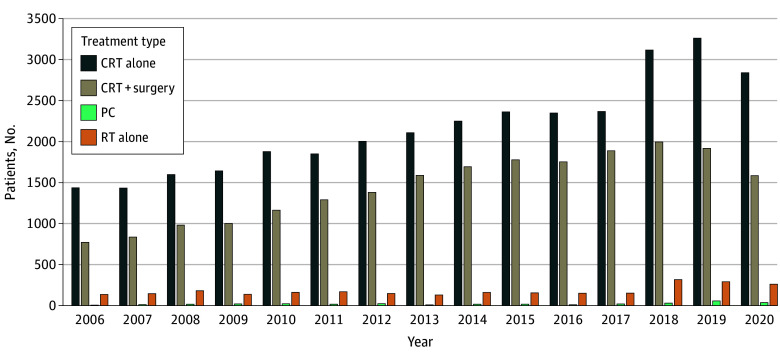
Treatment Modality Distribution Among Patients With Esophageal Cancer, 2006-2020 CRT indicates chemoradiation therapy; PC, perioperative chemotherapy; RT, radiotherapy.

In terms of treatment modalities received, 312 patients (0.5%) received perioperative chemotherapy, 283 (84.6%) of whom had EAC; 32 493 (56.9%) patients received DCRT, 21 619 (37.9%) received trimodality therapy, and 2692 (4.7%) received RT alone. Among patients who received perioperative chemotherapy, 290 (92.9%) received multiagent chemotherapy and 3 (1.0%) received single-agent chemotherapy; 9 patients (2.9%) did not receive chemotherapy due to high-risk factors. From 2006 to 2018, there was an overall increase in the number of patients receiving CRT plus surgical resection from 771 of 4351 (17.7%) in 2006 to 1995 of 7472 (26.7%) in 2018. There was a decrease in use of trimodality therapy with NCRT plus surgery from 2018 to 2020, with a gradual increase in the use of DCRT from 2019 to 2020. Overall, perioperative chemotherapy remained the most underused treatment modality from 2006 to 2020, with a gradual increase from 2016 to 2019 (Figure 1 and eTable 1 in Supplement 1).

### Survival Outcomes and Follow-Up

**Figure 2.**
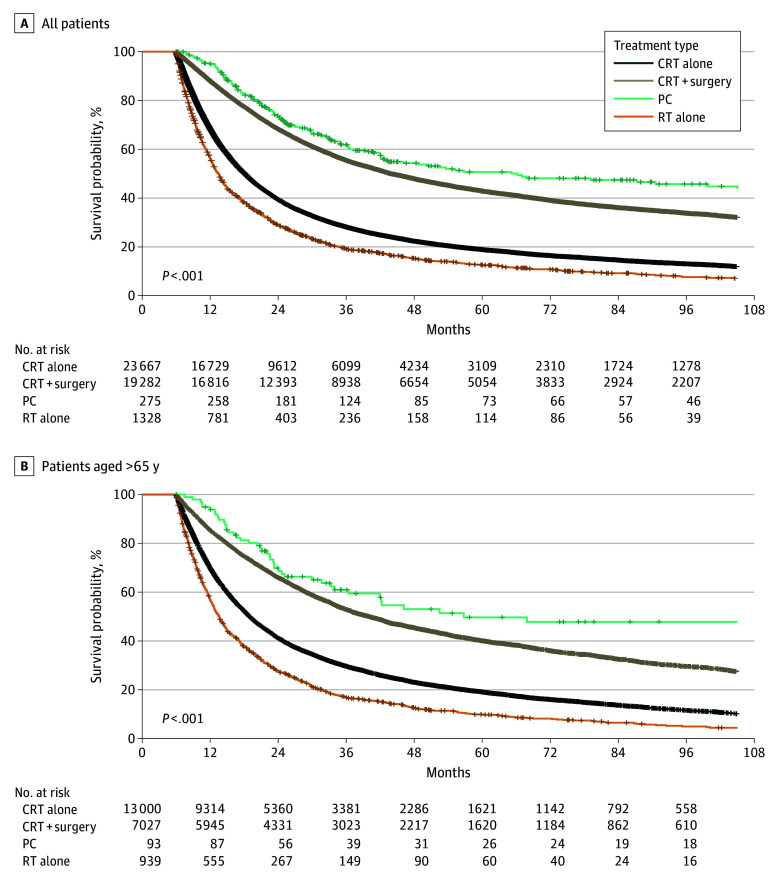
Overall Survival Among Patients With Esophageal Cancer by Treatment Modality CRT indicates chemoradiation therapy; PC, perioperative chemotherapy; RT, radiotherapy.

On multivariate survival analysis, the median OS among patients receiving perioperative chemotherapy (66.2 months; 95% CI, 43.1-111.9 months; *P* < .001) was longer compared with the median OS among patients receiving DCRT (18.1 months; 95% CI, 17.8-18.4 months; *P* < .001). Trimodality therapy was associated with a median OS of 43.9 months (95% CI, 42.8-45.5 months; *P* < .001), which was shorter than the OS among patients receiving perioperative chemotherapy but improved compared with OS among those receiving DCRT and RT alone. Radiotherapy alone was associated with a median OS of 13.5 months (95% CI, 12.8-14.0 months; *P* < .001). Similar results were found in the subgroup of patients with EAC, with a median OS of 56.7 months (95% CI, 42.1-07.4 months) in the perioperative chemotherapy group compared with 43.0 months (95% CI, 42.0-44.4 months; *P* < .001) in the trimodality group, 17.4 months (95% CI, 17.2-17.7 months; *P* < .001) in the DCRT group, and 13.7 months (95% CI, 13.0-14.7 months; *P* < .001) in the single-modality RT group. In the subgroup with ESCC, patients receiving CRT alone had a median OS of 20.1 months (95% CI, 19.4-20.7 months; *P* < .001), those receiving trimodality therapy had a median OS of 54.6 months (95% CI, 50.1-60.0 months; *P* < .001), and those receiving RT alone had a median OS of 12.8 months (95% CI, 12.0-13.7 months); the median OS for perioperative chemotherapy was not reached (Figure 2).

In multivariate adjusted survival analysis, perioperative chemotherapy (adjusted hazard ratio [AHR], 0.33; 95% CI, 0.28-0.39; *P* < .001) and trimodality therapy (AHR, 0.45; 95% CI, 0.44-0.46; *P* < .001) were associated with improved OS compared with DCRT. In contrast, RT alone was associated with worse outcomes compared with DCRT (AHR, 1.37; 95% CI, 1.30-1.45; *P* < .001). Patients with ESCC had worse survival outcomes compared with those with EAC (AHR, 0.87; 95% CI, 0.85-0.90; *P* < .001). In the overall population, worse performance status was associated with worse prognosis (CDCI score of 3 vs 0: AHR, 1.16; 95% CI, 1.09-1.24; *P* < .001). Table 2 details the full survival analysis.

**Table 2.** AHRs for All Patients and Histologic Subtypes

Variable	All patients		EAC		ESCC		Age >65 y	
	AHR (95% CI)	*P* value	AHR (95% CI)	*P* value	AHR (95% CI)	*P* value	AHR (95% CI)	*P* value
Age								
<50	1 [Reference]	NA	1 [Reference]	NA	1 [Reference]	NA	NA	NA
50-65	1.01 (0.97-1.05)	.73	0.98 (0.94-1.03)	.47	1.07 (0.98-1.16)	.13	NA	NA
≥65	1.02 (0.98-1.06)	.41	1.01 (0.96-1.06)	.66	1.05 (0.96-1.14)	.26	NA	NA
Treatment								
DCRT alone	1 [Reference]	NA	1 [Reference]	NA	1 [Reference]	NA	1 [Reference]	NA
NCRT and surgery	0.45 (0.44-0.46)	<.001	0.43 (0.42-0.44)	<.001	0.52 (0.50-0.55)	<.001	0.50 (0.48-0.52)	<.001
Perioperative chemotherapy	0.33 (0.28-0.39)	<.001	0.33 (0.28-0.40)	<.001	0.24 (0.09-0.65)	.005	0.32 (0.24-0.43)	<.001
RT alone	1.37 (1.30-1.45)	<.001	1.28 (1.19-1.37)	<.001	1.56 (1.43-1.70)	<.001	1.51 (1.41-1.61)	<.001
Histologic subtype								
EAC	1 [Reference]	NA	NA	NA	NA	NA	1 [Reference]	NA
ESCC	0.87 (0.85-0.90)	<.001	NA	NA	NA	NA	0.86 (0.82-0.89)	<.001
Not EAC or ESCC	0.99 (0.94-1.03)	.59	NA	NA	NA	NA	0.94 (0.88-1.00)	.06
Sex								
Female	0.86 (0.84-0.88)	<.001	0.89 (0.85-0.92)	<.001	0.85 (0.81-0.88)	<.001	0.89 (0.86-0.92)	<.001
Male	1 [Reference]	NA	1 [Reference]	NA	1 [Reference]	NA	Reference	NA
Race								
Black	1.10 (1.06-1.15)	<.001	1.03 (0.95-1.12)	.47	1.12 (1.07-1.17)	<.001	1.01 (0.95-1.08)	.70
East Asian, South Asian, or Pacific Islander	0.81 (0.75-0.88)	<.001	0.79 (0.69-0.91)	.001	0.81 (0.74-0.89)	<.001	0.86 (0.78-0.96)	.005
Hispanic	0.83 (0.78-0.88)	<.001	0.86 (0.79-0.92)	<.001	0.78 (0.71-0.86)	<.001	0.85 (0.77-0.93)	<.001
White	1 [Reference]	NA	1 [Reference]	NA	1 [Reference]	NA	1 [Reference]	NA
Other^a^	0.89 (0.82-0.98)	.02	0.93 (0.83-1.04)	.21	0.83 (0.70-0.97)	.02	0.88 (0.77-1.01)	.07
Grade								
Well differentiated	1 [Reference]	NA	1 [Reference]	NA	1 [Reference]	NA	1 [Reference]	NA
Moderately differentiated	1.18 (1.11-1.25)	<.001	1.10 (1.02-1.18)	.01	1.36 (1.23-1.50)	<.001	1.11 (1.03-1.21)	.01
Poorly differentiated	1.44 (1.36-1.52)	<.001	1.41 (1.32-1.51)	<.001	1.46 (1.33-1.62)	<.001	1.37 (1.26-1.48)	<.001
Unknown	1.16 (1.10-1.23)	<.001	1.12 (1.04-1.21)	.003	1.25 (1.13-1.39)	<.001	1.14 (1.05-1.24)	.002
CDCI score								
0	1 [Reference]	NA	1 [Reference]	NA	1 [Reference]	NA	1 [Reference]	NA
1	1.11 (1.08-1.13)	<.001	1.12 (1.09-1.16)	<.001	1.08 (1.02-1.13)	.004	1.11 (1.07-1.15)	<.001
2	1.18 (1.13-1.24)	<.001	1.15 (1.09-1.21)	<.001	1.27 (1.16-1.39)	<.001	1.24 (1.16-1.31)	<.001
3	1.16 (1.09-1.24)	<.001	1.16 (1.08-1.25)	<.001	1.19 (1.06-1.35)	.005	1.22 (1.12-1.32)	<.001

Abbreviations: AHR, adjusted hazard ratio; CDCI, Charlson-Deyo Comorbidity Index; DCRT, definitive chemoradiation therapy; EAC, esophageal adenocarcinoma; ESCC, esophageal squamous cell carcinoma; NA, not applicable; NCRT, neoadjuvant CRT; RT, radiotherapy.

^a^ Not further specified in the National Cancer Database.

In the subgroup analysis of patients with more than 6 months of follow-up who were older than 65 years, median follow-up was 77.0 months (IQR, 32.5-126.5 months) for perioperative chemotherapy, 67.8 months (IQR, 41.5-104.3 months) for trimodality therapy, 71.8 months (IQR, 41.9-116.5 months) for CRT alone, and 66.3 months (IQR, 41.6-106.6 months) for RT alone. In patients older than 65 years, those who received perioperative chemotherapy had a median OS of 56.7 months (95% CI, 36.4-115.2 months; *P* < .001), those receiving trimodality therapy had a median OS of 40.1 months (95% CI, 38.1-42.0 months; *P* < .001), those receiving DCRT had a median OS of 19.0 months (95% CI, 18.6-19.4 months; *P* < .001), and those receiving RT alone had a median OS of 13.6 months (95% CI, 12.8-14.4 months; *P* < .001). Figure 2 illustrates the subgroup analyses.

Median follow-up time for all patients was 73.7 months (IQR, 48.3-85.5 months). By treatment modality, median follow-up time was 74.5 months (IQR, 67.2-79.7 months) for perioperative chemotherapy, 73.5 months (IQR, 72.2-74.8 months) for trimodality therapy, 75.1 months (IQR, 73.2-76.5 months) for DCRT, and 74.5 months (IQR, 67.2-79.7 months) for RT alone.

## Discussion

In this study, tumor histologic subtype was associated with treatment outcomes among patients with locally advanced EC. Most patients who received perioperative chemotherapy had EAC, and these patients had the best survival rates compared with those receiving other treatment modalities. The Neo-AEGIS trial^7^ underscored a clinical equipoise between trimodality therapy and perioperative chemotherapy in managing EAC, while the ESOPEC trial^8^ provided evidence favoring perioperative chemotherapy for superior outcomes in EAC management. In this retrospective study, outcomes in the clinical setting aligned with the ESOPEC findings, showing improved survival among patients with EAC treated with perioperative chemotherapy. Trimodality therapy was also associated with improved survival compared with CRT or RT alone, with its efficacy already well established since the CROSS trial.^5^ The consistency of outcomes observed in the clinical setting supports the effectiveness of trimodality therapy in EC management. Notably, in all patients, perioperative chemotherapy was associated with improved outcomes compared with DCRT. However, consideration must be given to the patient population of this retrospective study, most of whom (66.0%) had EAC. Furthermore, tumor grade was associated with survival, with patients with poorly differentiated tumors experiencing the worst outcomes followed by those with moderately differentiated tumors.

Previous studies have indicated that patient age, independent of other factors, is associated with survival after surgery.^13^ With general life expectancy having improved in recent years, candidacy for surgery may vary among different institutions and may be largely affected by surgeons’ decisions. This factor should be carefully considered in clinical decision-making, particularly when determining whether esophagectomy should be recommended. Of note, at many academic centers, surgeons use geriatric assessments to evaluate the physiologic and functional status of patients to comprehensively determine risk instead of assessing age alone, especially for patients older than 65 years.^14^ The American College of Surgeons risk calculator is also routinely used to estimate risk based on a number of factors, including the type of surgery planned and patient comorbidities.^15^ In terms of age, the subgroup analysis of patients older than 65 years demonstrated overall worse survival outcomes in this group compared with patients younger than 50 years. For patients older than 65 years, trimodality treatment with NCRT followed by esophagectomy has been shown to be associated with improved survival outcomes compared with not undergoing surgical resection.^16^ Our study further supports these findings. In the ESOPEC trial,^8^ the median age of patients with EAC was 63 years, and the findings indicated that perioperative chemotherapy improved survival compared with neoadjuvant CROSS therapy. In our study, improved OS was seen among patients older than 65 years who received perioperative chemotherapy. Thus, even older patients should be counseled about the options of perioperative chemotherapy and NCRT followed by surgery. Age alone should not disqualify patients from multidisciplinary discussions about curative treatment approaches. However, in patients older than 65 years who are not candidates for surgical resection or who refuse surgical intervention, DCRT may be a reasonable option over single-modality RT.^17^ Our study also found improved OS outcomes associated with DCRT compared with RT alone. In addition, younger age and female sex were associated with more favorable treatment outcomes regardless of treatment modality. This finding aligns with existing literature.^18^

Studies have underscored the significance of socioeconomic status in influencing the outcomes and management of EC. Higher individual socioeconomic status correlates with lower 5-year mortality rates among patients with EC, whereas lower socioeconomic status is associated with diagnoses at older ages and more advanced disease stages.^19,20^ There is an urgent need to delve into the specific factors that shape survival outcomes in the context of socioeconomic status.^21^ When socioeconomic status was considered, Schlottmann et al^22^ found that factors such as insurance coverage, travel distance, income, and educational level were associated with the likelihood of undergoing surgical interventions and subsequent outcomes. In our study, a greater proportion of patients with private insurance received perioperative chemotherapy or trimodality therapy compared with those with public insurance. Studies have also shown that uninsured patients are less likely to receive comparable treatments than those with private insurance.^22^ Interestingly, patients traveling longer distances to receive treatment are more likely to be offered surgical resection with or without CRT.^22^ Notably, the majority of the patient cohort (69.3%) reported that they traveled up to a 31-km radius to access cancer treatment.

Additionally, patients with better performance status had improved response to treatment. This finding emphasizes the importance of overall health and functional well-being in influencing therapeutic efficacy. Improvements in survival outcomes may be related to the form of treatment modality patients receive based on their performance status. For example, patients with EC who had worse performance status were more likely to receive NCRT with surgery.^23^ Performance status is likely to be associated with survival outcomes because it is an important variable that determines treatments received.

### Limitations

A limitation of this study includes its retrospective design. In addition, the sample size of the perioperative chemotherapy group was small.

## Conclusions

In this cohort study of patients with stage II to III EC, perioperative chemotherapy and trimodality therapy were associated with improved survival outcomes compared with DCRT or single-modality RT. Notably, perioperative chemotherapy was underused and was associated with the greatest improvement in survival; however, the study population predominantly consisted of patients with EAC. This analysis revealed that patients older than 65 years also had survival benefit with perioperative chemotherapy and trimodality therapy. These findings demonstrate the evolving landscape of EC therapy and may help bridge the gap between clinical trial data and management and outcomes in the clinical setting.

## Notes

Supplement 1. **eTable 1.** Treatment Modality (Chemoradiation Therapy [CRT] Alone, CRT + Surgery, Perioperative Chemotherapy, Radiation Therapy [RT] Alone) Use From 2006 to 2020 **eTable 2.** Histology of Patients With Nonsquamous/Nonadenocarcinoma Esophageal Cancer **eTable 3.** Perioperative Chemotherapy Type

Supplement 2.
Data Sharing Statement

## References

[1] Sheikh M, Roshandel G, McCormack V, Malekzadeh R. Current status and future prospects for esophageal cancer. Cancers (Basel). 2023;15(3):765. 36765722 10.3390/cancers15030765 PMC9913274

[2] Harada K, Mizrak Kaya D, Baba H, Ajani JA. Recent advances in preoperative management of esophageal adenocarcinoma. F1000Res. 2017;6:501. 28491289 10.12688/f1000research.10794.1 PMC5399958

[3] Stahl M, Stuschke M, Lehmann N, . Chemoradiation with and without surgery in patients with locally advanced squamous cell carcinoma of the esophagus. J Clin Oncol. 2005;23(10):2310-2317. 15800321 10.1200/JCO.2005.00.034

[4] Bedenne L, Michel P, Bouché O, . Chemoradiation followed by surgery compared with chemoradiation alone in squamous cancer of the esophagus: FFCD 9102. J Clin Oncol. 2007;25(10):1160-1168. 17401004 10.1200/JCO.2005.04.7118

[5] van Hagen P, Hulshof MCCM, van Lanschot JJB, ; CROSS Group. Preoperative chemoradiotherapy for esophageal or junctional cancer. N Engl J Med. 2012;366(22):2074-2084. 22646630 10.1056/NEJMoa1112088

[6] Eyck BM, van Lanschot JJB, Hulshof MCCM, ; CROSS Study Group. Ten-year outcome of neoadjuvant chemoradiotherapy plus surgery for esophageal cancer: the randomized controlled CROSS trial. J Clin Oncol. 2021;39(18):1995-2004. 33891478 10.1200/JCO.20.03614

[7] Reynolds JV, Preston SR, O’Neill B, ; Neo-AEGIS Investigators and Trial Group. Trimodality therapy versus perioperative chemotherapy in the management of locally advanced adenocarcinoma of the oesophagus and oesophagogastric junction (Neo-AEGIS): an open-label, randomised, phase 3 trial. Lancet Gastroenterol Hepatol. 2023;8(11):1015-1027. 37734399 10.1016/S2468-1253(23)00243-1 PMC10567579

[8] Hoeppner J, Brunner T, Lordick F, . Prospective randomized multicenter phase III trial comparing perioperative chemotherapy (FLOT protocol) to neoadjuvant chemoradiation (CROSS protocol) in patients with adenocarcinoma of the esophagus (ESOPEC trial). J Clin Oncol. 2024;42(17 suppl):LBA1. 10.1186/s12885-016-2564-y PMC4952147 27435280

[9] Park D, Jeon WJ, Yang C, Castillo DR. Advancing esophageal cancer treatment: immunotherapy in neoadjuvant and adjuvant settings. Cancers (Basel). 2024;16(2):318. 38254805 10.3390/cancers16020318 PMC10813716

[10] Chen Z, Ren Y, Du XL, . Incidence and survival differences in esophageal cancer among ethnic groups in the United States. Oncotarget. 2017;8(29):47037-47051. 28410201 10.18632/oncotarget.16694 PMC5564542

[11] Charlson ME, Carrozzino D, Guidi J, Patierno C. Charlson Comorbidity Index: a critical review of clinimetric properties. Psychother Psychosom. 2022;91(1):8-35. 34991091 10.1159/000521288

[12] Schemper M, Smith TL. A note on quantifying follow-up in studies of failure time. Control Clin Trials. 1996;17(4):343-346. 8889347 10.1016/0197-2456(96)00075-x

[13] Kauppila JH, Mattsson F, Lagergren J. Impact of age on the treatment and survival in esophagogastric cancer. Ann Surg Oncol. 2023;30(5):2716-2725. 36648617 10.1245/s10434-022-13052-4 PMC10085923

[14] Sun V, Raz DJ, Kim JY, . Barriers and facilitators of adherence to a perioperative physical activity intervention for older adults with cancer and their family caregivers. J Geriatr Oncol. 2020;11(2):256-262. 31208829 10.1016/j.jgo.2019.06.003 PMC6911031

[15] Bilimoria KY, Liu Y, Paruch JL, . Development and evaluation of the universal ACS NSQIP surgical risk calculator: a decision aid and informed consent tool for patients and surgeons. J Am Coll Surg. 2013;217(5):833-42.e1, 3. 24055383 10.1016/j.jamcollsurg.2013.07.385 PMC3805776

[16] Rahimy E, Koong A, Toesca D, . Outcomes and tolerability of definitive and preoperative chemoradiation in elderly patients with esophageal cancer: a retrospective institutional review. Adv Radiat Oncol. 2020;5(6):1188-1196. 33305080 10.1016/j.adro.2020.05.001 PMC7718494

[17] Derby S, Forshaw M, Lowrie C, . Single modality radical radiotherapy is an acceptable alternative for the older patient with squamous cell carcinoma of the oesophagus. BMJ Open Gastroenterol. 2021;8(1):e000492. 33504498 10.1136/bmjgast-2020-000492 PMC7843319

[18] Xiang ZF, Xiong HC, Hu DF, . Age-related sex disparities in esophageal cancer survival: a population-based study in the United States. Front Public Health. 2022;10:836914. 35903385 10.3389/fpubh.2022.836914 PMC9314568

[19] Wu CC, Chang CM, Hsu TW, . The effect of individual and neighborhood socioeconomic status on esophageal cancer survival in working-age patients in Taiwan. Medicine (Baltimore). 2016;95(27):e4140. 27399129 10.1097/MD.0000000000004140 PMC5058858

[20] Bus P, Aarts MJ, Lemmens VEPP, . The effect of socioeconomic status on staging and treatment decisions in esophageal cancer. J Clin Gastroenterol. 2012;46(10):833-839. 22460163 10.1097/MCG.0b013e31824e8ff8

[21] Xie SH, Lagergren J. Social group disparities in the incidence and prognosis of oesophageal cancer. United European Gastroenterol J. 2018;6(3):343-348. 29774147 10.1177/2050640617751254 PMC5949978

[22] Schlottmann F, Gaber C, Strassle PD, Herbella FAM, Molena D, Patti MG. Disparities in esophageal cancer: less treatment, less surgical resection, and poorer survival in disadvantaged patients. Dis Esophagus. 2020;33(2):doz045. 31076759 10.1093/dote/doz045 PMC8205620

[23] Kitti PM, Faltinova M, Kauppi J, . Chemoradiation for oesophageal cancer: the choice of treatment modality. Radiat Oncol. 2023;18(1):93. 37259100 10.1186/s13014-023-02290-9 PMC10230716

