# Supplementary material for: Survival and Treatment Patterns in Stage II to III Esophageal Cancer
Source: JAMA Netw Open. 2024 Oct 21;7(10):e2440568. doi: 10.1001/jamanetworkopen.2024.40568 (PMC11581628; doi:10.1001/jamanetworkopen.2024.40568)
Supplement: Supplement 1. — eTable 1. Treatment Modality (Chemoradiation Therapy [CRT] Alone, CRT + Surgery, Perioperative Chemotherapy, Radiation Therapy [RT] Alone) Use From 2006 to 2020 eTable 2. Histology of Patients With Nonsquamous/Nonadenocarcinoma Esophageal Cancer eTable 3. Perioperative Chemotherapy Type [file jamanetwopen-e2440568-s001.pdf]

Supplementary Online Content

Jeon WJ, Park D, Al-Manaseer F, et al. Survival and treatment patterns in stage II to III esophageal cancer. *JAMA Netw Open*. 2024;7(10):e2440568. doi:10.1001/jamanetworkopen.2024.40568

eTable 1. Treatment Modality (Chemoradiation Therapy [CRT] Alone, CRT + Surgery, Perioperative Chemotherapy, Radiation Therapy [RT] Alone) Use From 2006 to 2020

eTable 2. Histology of Patients With Nonsquamous/Nonadenocarcinoma Esophageal Cancer

eTable 3. Perioperative Chemotherapy Type

This supplementary material has been provided by the authors to give readers additional information about their work.

eTable 1. Treatment Modality (Chemoradiation Therapy [CRT] Alone, CRT + Surgery, Perioperative Chemotherapy, Radiation Therapy [RT] Alone) Use From 2006 to 2020

Year	CRT Alone	CRT + Surgery	Perioperative Chemotherapy	RT Alone
**2006**	1436	771	5	136
**2007**	1433	835	12	145
**2008**	1598	981	16	181
**2009**	1642	1001	20	137
**2010**	1878	1164	22	161
**2011**	1850	1290	17	169
**2012**	2004	1381	24	147
**2013**	2107	1589	9	130
**2014**	2249	1693	17	160
**2015**	2363	1777	18	155
**2016**	2348	1753	10	150
**2017**	2367	1888	19	152
**2018**	3117	1995	30	317
**2019**	3261	1916	57	291
**2020**	2840	1585	36	261

© 2024 Jeon WJ et al. *JAMA Network Open.*

eTable 2. Histology of Patients With Nonsquamous/Nonadenocarcinoma Esophageal Cancer		
**Code**	**Count**	**Histology Description**
8000/3	84	Neoplasm, malignant
8004/3	1	Malignant tumor, spindle cell type
8010/3	674	Carcinoma, NOS
8012/3	10	Large cell carcinoma, NOS
8013/3	36	Large cell neuroendocrine carcinoma
8014/3	1	Large cell carcinoma with rhabdoid phenotype
8020/3	19	Carcinoma, undifferentiated type, NOS
8021/3	4	Carcinoma, anaplastic type, NOS
8022/3	1	Pleomorphic carcinoma
8032/3	7	Spindle cell carcinoma
8033/3	19	Pseudosarcomatous carcinoma
8041/3	204	Small cell carcinoma, NOS
8042/3	1	Small cell carcinoma, fusiform cell
8045/3	13	Combined small cell carcinoma
8046/3	53	Non-small cell carcinoma, NOS
8051/3	13	Verrucous carcinoma, NOS
8052/3	6	Papillary squamous cell carcinoma
8072/3	154	Squamous cell carcinoma, lg. cell, non-ker.
8073/3	5	Sq. cell carcinoma, sm. cell, non-ker.
8074/3	32	Sq. cell carcinoma, spindle cell
8075/3	2	Squamous cell carcinoma, adenoid
8076/3	4	Sq. cell carcinoma, micro-invasive

© 2024 Jeon WJ et al. *JAMA Network Open.*

8078/3	1	Sq. cell carcinoma with horn formation
8082/3	1	Lymphoepithelial carcinoma
8083/3	1	Basaloid squamous cell carcinoma
8094/3	97	Basal cell carcinoma, NOS
8123/3	5	Transitional cell carcinoma, spindle cell
8141/3	4	Scirrhous adenocarcinoma
8160/3	1	Hepatocellular carcinoma, NOS
8190/3	1	Endometrioid carcinoma, NOS
8200/3	5	Adenoid cystic carcinoma
8210/3	8	Cribriform carcinoma
8211/3	10	Adenocarcinoma in villous adenoma
8244/3	25	Composite carcinoid
8246/3	140	Neuroendocrine carcinoma
8249/3	2	Neuroendocrine tumor
8260/3	50	Papillary adenocarcinoma, NOS
8261/3	3	Adenocarcinoma in villous adenoma
8263/3	8	Adenocarcinoma in tubulovillous adenoma
8310/3	3	Clear cell adenocarcinoma, NOS
8323/3	11	Mixed cell adenocarcinoma
8430/3	3	Mucoepidermoid carcinoma
8480/3	455	Mucinous adenocarcinoma
8481/3	109	Mucin-producing adenocarcinoma, NOS
8482/3	1	Signet ring cell carcinoma
8530/3	2	Adenosquamous carcinoma, NOS
8560/3	337	Adenosquamous carcinoma
8570/3	3	Adenocarcinoma with squamous metaplasia

© 2024 Jeon WJ et al. *JAMA Network Open.*

8574/3	65	Adenocarcinoma with neuroendocrine differentiation
8576/3	1	Undifferentiated carcinoma, spindle cell
8800/3	1	Sarcoma, NOS
8890/3	3	Leiomyosarcoma, NOS
8936/3	1	Stromal sarcoma, NOS
8980/3	6	Carcinosarcoma, NOS
9120/3	1	Hemangiosarcoma
**Legend**: NOS: Not otherwise specified, Sq: squamous		

© 2024 Jeon WJ et al. *JAMA Network Open.*

eTable 3. Perioperative Chemotherapy Type

**Perioperative Chemotherapy Type**	
No Chemotherapy Given Due to High Risk Factors	9
Chemotherapy: Multi-Agent	290
Chemotherapy: Single-Agent	3

© 2024 Jeon WJ et al. *JAMA Network Open.*
