# Supplementary material for: Survival and Treatment Patterns in Stage II to III Esophageal Cancer
Source: JAMA Netw Open. 2024 Oct 21;7(10):e2440568. doi: 10.1001/jamanetworkopen.2024.40568 (PMC11581628; doi:10.1001/jamanetworkopen.2024.40568)
Supplement: Supplement 2. — Data Sharing Statement [file jamanetwopen-e2440568-s002.pdf]

Data Sharing Statement

Jeon. Survival and Treatment Patterns in Stage II to III Esophageal Cancer. *JAMA Netw Open*. Published October 22, 2024. doi:10.1001/jamanetworkopen.2024.40568

Data
Data available: No
